# Predicting the main pollen season of *Broussonetia Papyrifera* (paper mulberry) tree

**DOI:** 10.1371/journal.pone.0296878

**Published:** 2024-02-02

**Authors:** Ahmad Kakakhail, Aimal Rextin, Jeroen Buters, Chun Lin, José M. Maya-Manzano, Mehwish Nasim, Jose Oteros, Antonio Picornell, Hillary Pinnock, Jurgen Schwarze, Osman Yusuf

**Affiliations:** 1 The Allergy & Asthma Institute, Islamabad, Pakistan; 2 Department of Computer Science, National University of Modern Languages, Rawalpindi, Pakistan; 3 National University of Science and Technology, Islamabad, Pakistan; 4 Center of Allergy & Environment (ZAUM), Member of the German Center for Lung Research (DZL), Technical University and Helmholtz Center, Munich, Germany; 5 NIHR Global Health Research Unit on Respiratory Health (RESPIRE), Usher Institute, The University of Edinburgh, Edinburgh, United Kingdom; 6 Department of Plant Biology, Ecology and Earth Sciences, University of Extremadura, Badajoz, Spain; 7 Flinders University, Adelaide, Australia; 8 The University of Western Australia, Perth, Australia; 9 University of Córdoba, Córdoba, Spain; 10 Department of Botany and Plant Physiology, University of Malaga, Málaga, Spain; 11 Child Life and Health, Centre for Inflammation Research, Queen’s Medical Research Institute, The University of Edinburgh, Edinburgh, United Kingdom; Satyawati College, University of Delhi, INDIA

## Abstract

Paper mulberry pollen, declared a pest in several countries including Pakistan, can trigger severe allergies and cause asthma attacks. We aimed to develop an algorithm that could accurately predict high pollen days to underpin an alert system that would allow patients to take timely precautionary measures. We developed and validated two prediction models that take historical pollen and weather data as their input to predict the start date and peak date of the pollen season in Islamabad, the capital city of Pakistan. The first model is based on linear regression and the second one is based on phenological modelling. We tested our models on an original and comprehensive dataset from Islamabad. The mean absolute errors (MAEs) for the *start* day are 2.3 and 3.7 days for the linear and phenological models, respectively, while for the *peak* day, the MAEs are 3.3 and 4.0 days, respectively. These encouraging results could be used in a website or app to notify patients and healthcare providers to start preparing for the paper mulberry pollen season. Timely action could reduce the burden of symptoms, mitigate the risk of acute attacks and potentially prevent deaths due to acute pollen-induced allergy.

## Introduction

**Fig 1:**
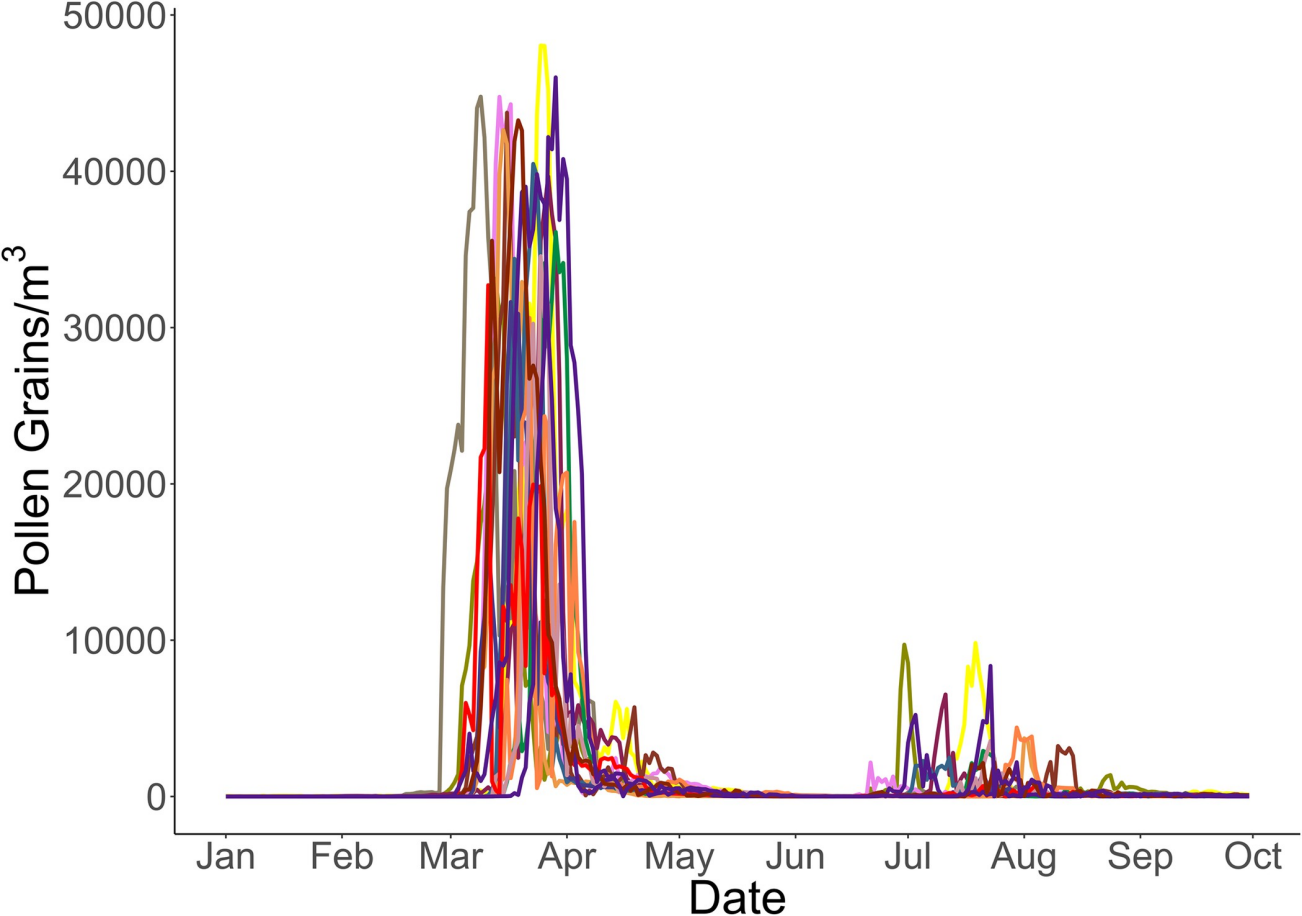
Annual paper mulberry pollen count time-series for the years 2004–2018. Each line represents a separate year. The main pollen seasons (periods with the highest pollen concentrations) are recorded between 10^th^ and 31^st^ March. Pollen concentrations rise again slightly between July and August during and after the summer rain.

Paper Mulberry (*Broussonetia papyrifera* (L.) L’Hér. ex Vent) is a native tree of Japan and China and is 3–12 m tall. It is a small quick-growing tree with red to orange fruits. Each inflorescence of paper mulberry can yield approximately 150–200 catkins [1], resulting in extremely high pollen concentrations (40,000 pollen grains/m^3^), for a short period every spring [2] (Fig 1). It is an invasive plant and is considered a pest in various countries including Argentina and Pakistan. In some Australian states such as Western Australia, it is declared a prohibited plant, and only research organisations can keep it subject to a permit. In Queensland, this tree is sparingly naturalised around the capital city of Brisbane and the coastal wet tropics. However, authorities are aware that it can become a significant problem if its spread is not controlled. Paper mulberry was initially planted for ornamental purposes in Islamabad, but due to its invasive nature, it has spread widely [3] and is now difficult to eradicate despite serious efforts made by government agencies [4]. The invasive nature of paper mulberry is not the only problem with this plant. Severe allergic reactions from the pollen of this tree have been reported in the literature.

The severity of the allergic or asthmatic pollen reactions varies according to individual clinical status and also depends upon the surrounding environment as pollen allergenicity can differ by location [5]. The high level of paper mulberry pollen in spring can cause severe asthmatic reactions [6]. The World Health Organisation (WHO) estimates that it causes more than 40% of all respiratory allergies in Islamabad, including symptoms of allergic rhinoconjunctivitis and asthma, which sometimes require hospitalisation [7]. Therefore, there is a need to predict the onset of pollen seasons to inform those affected to take preventive measures beforehand [8, 9]. With recent advances in data mining techniques [10] and an increase in the computational power of even small handheld devices, very precise estimates can be provided by using such prediction algorithms.

**Fig 2:**
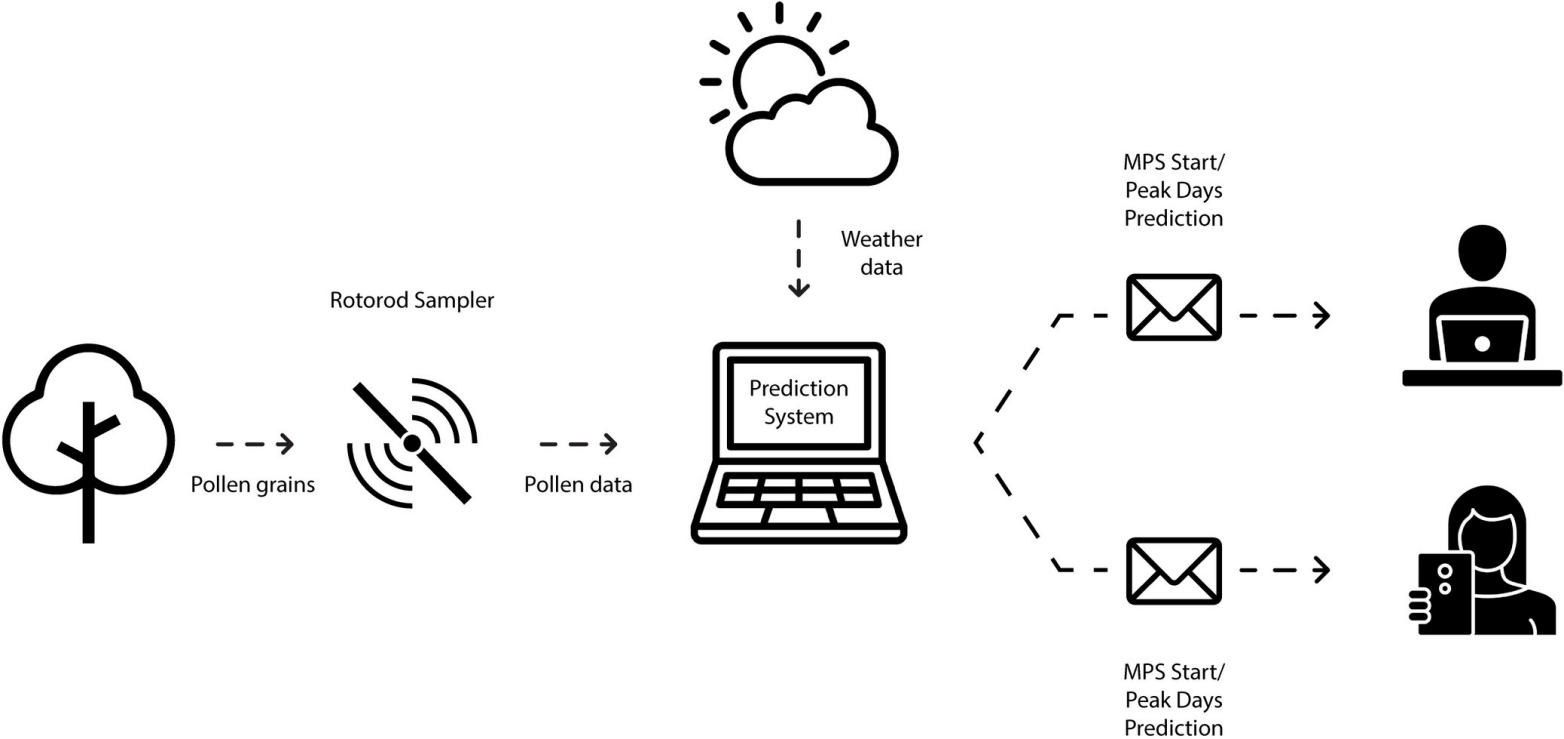
Pictorial representation of the pollen prediction system. Here MPS denotes the Main Pollen Season (the time of the year with the highest pollen concentration).

This paper aims to use historical pollen concentrations along with meteorological data to predict when pollen concentrations will reach levels that are likely to cause allergy and asthma symptoms in patients. A schematic of such a system is shown in Fig 2. This will allow patients or their caregivers to take timely preventive measures, e.g., to start required medication, to stay indoors or to move temporarily to a dry mountain climate to reduce or avoid severe pollen allergies [11]. Such a prediction system could then be implemented on a server that constantly gathers daily weather and pollen concentrations to inform the prediction. It is expected that these forecasts will be more accurate than static pollen calendars that are generally constructed by taking the mean dates of historical pollen seasons [8].

## Materials and methods

### Datasets

In this work, we demonstrate two forecasting models for pollen concentration of paper mulberry. Though we use a dataset from Islamabad where this plant has become a weed, we believe our models will be useful in other countries as well. We obtained daily pollen concentrations from the Pakistan Meteorological Department (PMD) website [12] at the end of 2019. PMD reports daily concentrations of eight pollen types including Paper Mulberry on their website. We used data from 1^st^ January 2004 to 31^st^ December 2018 to ensure that we had complete data for each calendar year. These pollen samples had been collected using a single rotorod sampler in Islamabad. After counting under a microscope the pollen particles in daily samples, pollen concentrations were reported by PMD as particles per cubic meter.

Simulated weather data were obtained from www.meteoblue.com, because weather data from PMD [12] had a significant number of missing values. We analysed the mean temperatures of both simulated and observed weather data by computing its absolute differences for the first four months of all the years from 2003–2019, and found the mean absolute difference of 1.7°C. This shows the reliability of the simulated weather data with respect to the observed data from PMD. Only the first four months were used to evaluate the reliability of the simulated weather data as these were the only weather data used in our models. The initial weather variables are observations from national weather services, which are numerically imputed for missing values. This type of hybrid weather data has been used in studies when weather information is incomplete [13] or when the effect of topology on weather needs to be considered [14].

### Evaluation

The dataset was divided into two parts: the initial twelve years from 1^st^ January 2004 to 31^st^ December 2015 (80% of the data) were used as the training set, while the last three years from 1^st^ January 2016 to 31^st^ December 2018 (20% of the data) were the test set.

The performance of each model was assessed by calculating the mean absolute error (MAE) using the test set:

$$MAE=\frac{\sum_{i=1}^{n}\left|PD_{i}-AD_{i}\right|}{n}$$ (1)

Here, *PD*_*i*_ and *AD*_*i*_ are predicted and actual pollen season dates respectively for Year *i*. A lower MAE indicates a more accurate model.

### Algorithmic approaches

We aimed to predict the following:

1. Start Day: The day when 5% [15, 16] or 2.5% [17, 18] of the annual pollen has accumulated.
2. Peak Day: The day with the highest pollen count for a particular year.

Notably, the start day is defined in two different ways in literature, which will be referred to as the two criteria of the start day.

Since on average the pollen season starts on 8^th^ March, we give our first prediction on 25^th^ February and then update this prediction every day by incorporating more observed data. Weather variables required for predicting pollen seasons after 25^th^ February were estimated by combining historical observed weather data for that date. Because short-term weather forecasts for a small area are more accurate, a weighted average was calculated with 80% weight given to the actual data and 20% weight given to the mean value of the weather parameters on that date in the historical data (from the training years).

#### Regression models

Linear Regression (LR) is an algorithm that finds the best possible linear fit between one or more explanatory variables and a response variable by fitting a linear equation to the observed data. The linear model has the following form:

$$y=a+\sum_{i=1}^{n}b_{i}x_{i}$$ (2)

Here *x*_1_, *x*_2_, ···, *x*_*n*_ are *n* ≥ 1 explanatory variables and *y* is the response variable; *a* is the intercept, whereas *b*_*i*_ is the slope associated with *x*_*i*_. The linear regression algorithm finds the best possible values of *a* and *b*_1_, *b*_2_, ···, *b*_*n*_. Regression based models using various weather parameters and historical pollen concentrations have been applied to predict pollen seasons [2, 10].

Linear regression needs to be applied to various durations of weather data in the training set to select the optimal duration as input to the final model. For this purpose, all possible durations with the start date varying from 1^st^ January to 25^th^ February and the end date varying from 1^st^ March to 11^th^ March were tried for the start day prediction. However, in case of the peak day prediction, all possible durations with the start date varying from 1^st^ January to 25^th^ February were tried while the end date was fixed at the start date of the main pollen season. In each iteration, the best subset of predictor variables was identified and then linear regression was applied along with cross validation to the identified set of variables. The detail of these two steps is given below:

*Step 1*: *Variable selection*. The following meteorological factors were considered potential predictor variables for the pollen season dates:

- Temperature as *T*
- Humidity as *H*
- Cloud Cover as *C*_*c*_
- Sunshine Duration as *S*_*d*_
- Wind Speed as *W*_*s*_
- Wind Direction as *W*_*d*_
- Wind Gust as *W*_*g*_
- Rainfall as *R*_*f*_

However, a subset of the most influential weather variables needed to be identified, as inter-correlated variables could lead to overfitting of the model and degrade its predictive ability on unseen data. For the purpose of parameter selection, we used a stepwise variable selection technique (for each duration) which adds one variable at a time and selects the one with the highest adjusted *R*^2^. Here adjusted *R*^2^ instead of *R*^2^ was used because it is normalized with each additional variable reducing the risk of model over-fitting [19]. This method works in the following way:

1. In the first iteration, linear regression was applied to each of the variables and the single variable that resulted in a linear regression model with the highest *R*^2^ (the goodness of fit) was selected.
2. In the following *k* iterations, linear regression was applied to all subsets of *k* variables, before a *k*-independent-variable linear regression model with the highest adjusted *R*^2^ was selected.
3. In the end, among the *p* regression models, where *p* was the initial number of variables, we selected the one with the highest adjusted *R*^2^.

*Step 2*: *Cross validation*. We used the Leave-One-Out-Cross-Validation (LOOCV) variant of *k*-fold cross validation [20] to check the robustness of the fitted models. It left aside one training year and trains the model for the remaining *n* − 1 years, and then evaluated the model on the left-out year. This process was repeated by leaving out each year in the training set one by one. The final linear model was obtained by averaging the coefficients of the linear models derived in the various iterations of LOOCV, and MAE on this aggregated model was also computed. To implement LOOCV, the R library caret was used.

After performing these two steps, all linear models consisting of the most influential predictor variables for each possible duration and their associated MAE were evaluated. Models with *p*-value greater than 0.05 were discarded as they were unlikely to fit well on new data in the test years. Linear models with the minimum MAE were chosen.

#### Phenological models

Phenological models predict pollen seasons based on the amount of heat that the plants need to start the flowering phenophase [16, 21–23]. In this paper, these required heat units are interchangeably called forcing units and simply heat units.

The start day of the pollen season was predicted using the equation below [16]:

$$s_{f}=\sum_{F_{1}}^{F}\left(\frac{1}{1+e^{d(t_{avg}-c)}}\right)$$ (3)

Here, *sf* represents the accumulated heat needed for paper mulberry to flower, *F*_1_ represents the date when heat accumulation for pollination starts. *F is* the start day of the main pollen season, i.e., when enough heat is accumulated for pollination to start. *d* is a numeric parameter with a negative value for normalising the resulting accumulated heat units. *t*_*avg*_ is the daily average temperature, while *c* represents the temperature threshold, i.e., only when the average daily temperature crosses *c* does heat contributes to the start of pollination.

The peak day was predicted using the following equation [16]:

$$Heat=\sum_{D_{T}}^{D}\left(T_{avg}-T_{h}\right)$$ (4)

Here *D*_*T*_ represents the first day after the pollen season’s start day when the average temperature crosses a threshold *T*_*D*_, and *D* is the peak day of the pollen season; *T*_*avg*_ is the daily average temperature, and *T*_*h*_ is the temperature threshold that needs to be surpassed for a particular day for heat to accumulate.

However, the challenge was that all parameters of these equations except the daily average temperature *t*_*avg*_ were unknown, including the required heat units. Therefore, a brute force approach was used to try all possible values of the unknown parameters and pick the combination yielding the consistent forcing units over the years, specific to paper mulberry. The best combination was determined based on the training data only and the test data were reserved to evaluate the prediction performance.

The best combination of parameters was determined by first generating all possible combinations of values for the parameters *c*, *d* and *F*_1_ (for the start day prediction) or *T*_*D*_ and *T*_*h*_ (for the peak day prediction). More specifically, for *F*_1_, we used the dates from 1^st^ October to 28^th^ February; for *d*, we used values between −10 and 0, with an increment of 0.25. We used temperatures between 0°C and 40°C with an increment of 1°C for *c*, *T*_*D*_ and *T*_*h*_. Then each combination of these parameters was applied to Eq 3 or Eq 4 to calculate the forcing units for each year. The mean and standard deviation (SD) of the derived forcing units across the years were then calculated for each combination of parameters. The mean forcing units for each combination of parameters was denoted as the candidate heat units for the combination.

The combinations of parameters resulting in candidate forcing units of 0 were discarded, as the forcing units should be non-zero to start the pollination process. Secondly, the combinations resulting in relative SDs larger than 50% were also discarded, large RSDs indicate greater uncertainty in estimating the plant-specific heat units. The final combination of parameters was determined within the remaining combinations based on the model MAE.

## Results

### Linear regression models

#### Start day prediction

**Fig 3:**
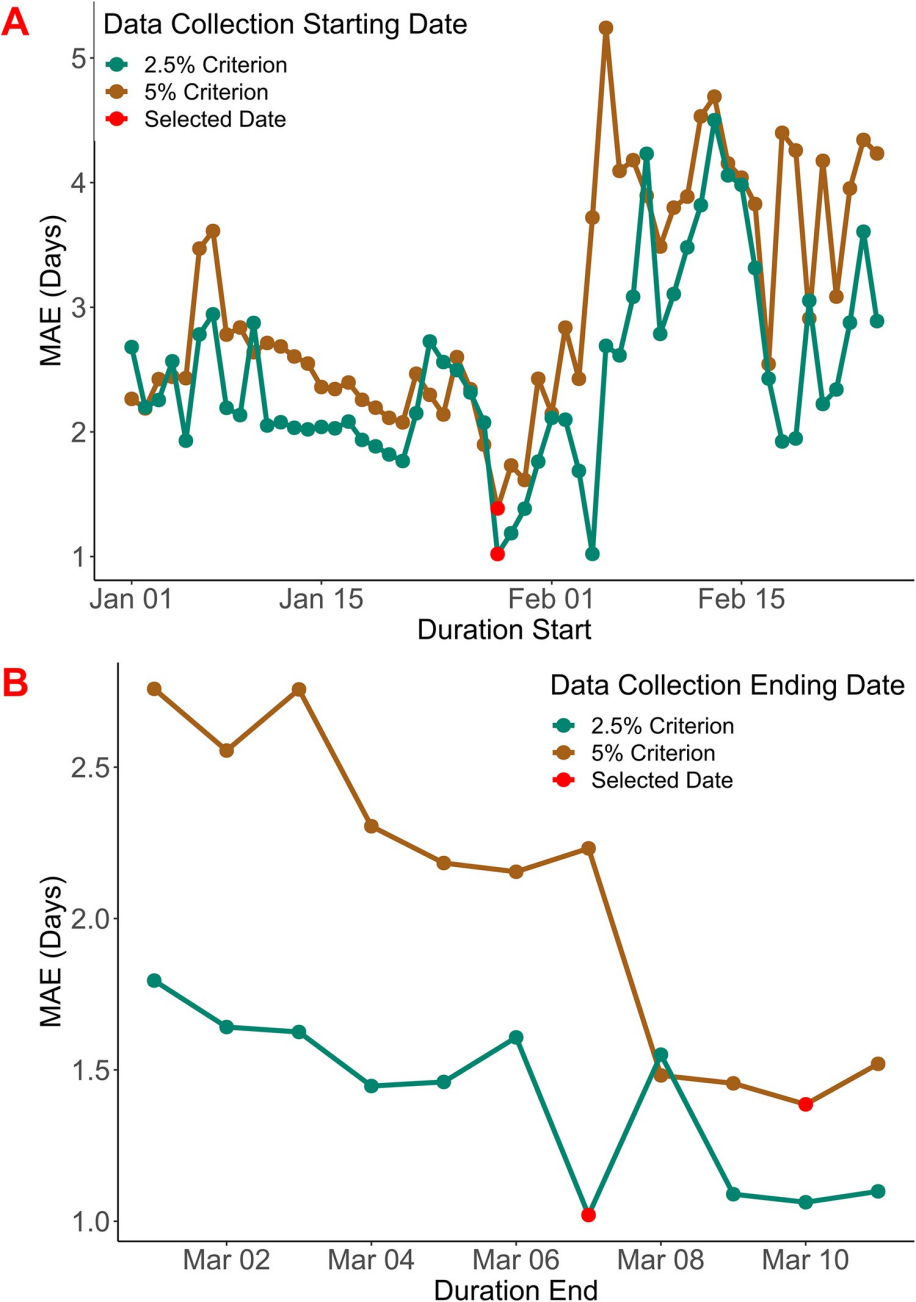
Mean Absolute Error (MAE) calculated over various time intervals using their corresponding meteorological data for the start day prediction under the 2.5% and 5% start day criteria: (A) the various starting points (B) the various ending points. The number of total combinations considered in this brute force optimization was 11 × 56. Only the combinations of starting points yielding the best ending points (and vice versa) are shown. This was only done for the training years as the testing years were reserved to evaluate the performance of the algorithm.

For the 5% start day criterion, the most accurate prediction was obtained when the weather data from 28^th^ January to 10^th^ March were used, giving a MAE of 1.4 days (Fig 3). The model identified seven most influential predictor variables. The complete linear model (*P*_*s*5_) for the start day prediction under the 5% start day criterion is:

$$P_{s5}=177.358-4.409\times T-0.491\times H+0.011\times R_{f}-0.391\times C_{c}-0.098\times S_{d}+0.154\times W_{d}+2.186\times W_{g}$$ (5)

In the case of the 2.5% start day criterion, six predictor variables were identified for the optimal period between 28^th^ January and 7^th^ March, with a model MAE of 1.0 day (Fig 3). The complete linear model (*P*_*s*2.5_) for the start day prediction under the 2.5% start day criterion is:

$$P_{s2.5}=137.151-3.931\times T-0.570\times H+0.010\times R_{f}-0.053\times S_{d}+0.170\times W_{d}+2.282\times W_{g}$$ (6)

Data from the testing years were used to evaluate the performance of these two models on unseen data. The MAE for the start day prediction under both criteria was 2.3 days.

#### Peak day prediction

**Fig 4:**
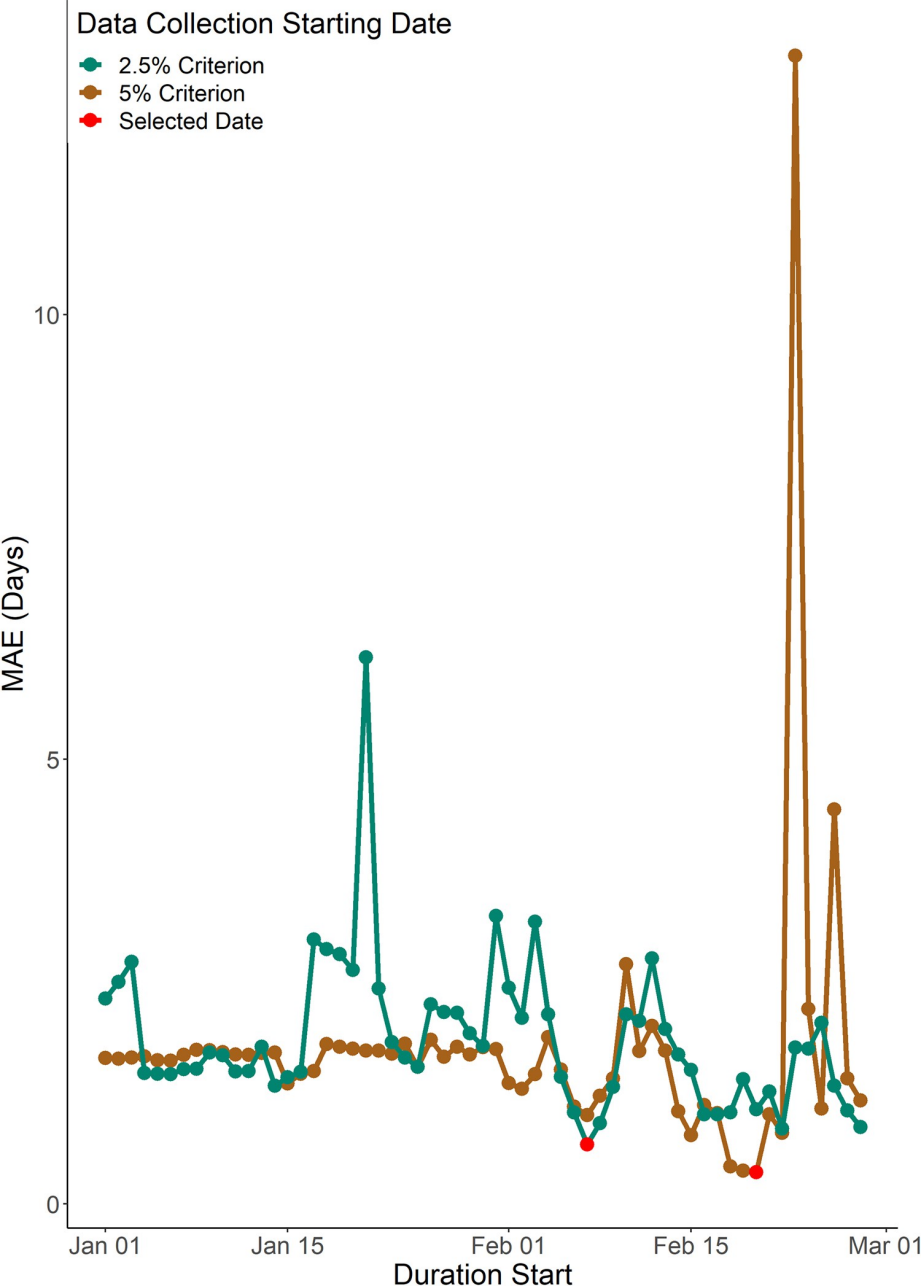
Mean Absolute Error (MAE) values for various durations of meteorological data for the peak day prediction under the 2.5% and 5% start day criteria. This was done only for the training years as the testing years were reserved to evaluate the performance of the algorithm.

Under the 5% peak day criterion the best prediction was obtained for the duration starting from 20^th^ February with a resulting MAE of 0.4 day (Fig 4). Eight influential predictor variables were identified for this optimal period. The complete linear model (*P*_*p*5_) for peak day prediction under the 5% start day criterion is:

$$P_{p5}=90.623-0.301\times P_{s5}-1.183\times T+0.016\times H-0.187\times C_{c}-0.031\times S_{d}-1.039\times W_{s}+0.371\times W_{d}-0.126\times W_{g}$$ (7)

In case of the 2.5% start day criterion, seven best predictor variables were identified for the optimal period starting from 7^th^ February with a resulting MAE of 0.7 day (Fig 4). The linear model for peak day prediction under the 2.5% start day criterion is:

$$P_{p2.5}=97.853+0.635\times P_{s2.5}-1.249\times T-0.026\times R_{f}-0.534\times C_{c}-0.111\times S_{d}+0.138\times W_{d}+1.105\times W_{g}$$ (8)

Evaluation of these linear models using data from the testing years resulted in an MAE of 3.3 days under the 5% start day criterion and an MAE of 3.7 days under the 2.5% start day criterion.

#### Phenological models

**Fig 5:**
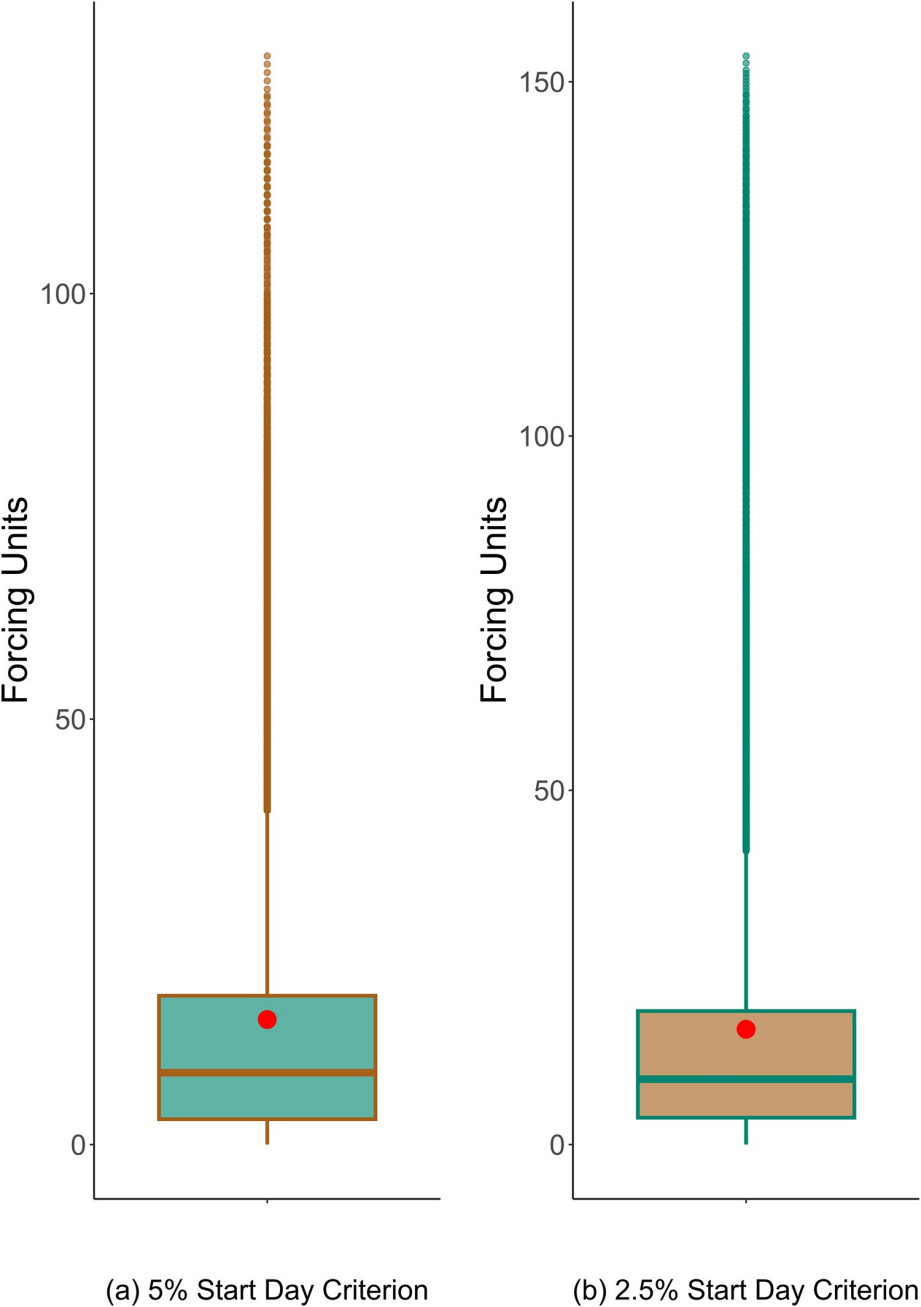
The box plot of candidate heat units for the start day after all filtration has been performed including removal of the entries whose relative standard deviation is higher than 50% across years. The red dots show *M*, the mean values that will be used as the required heat units.

**Fig 6:**
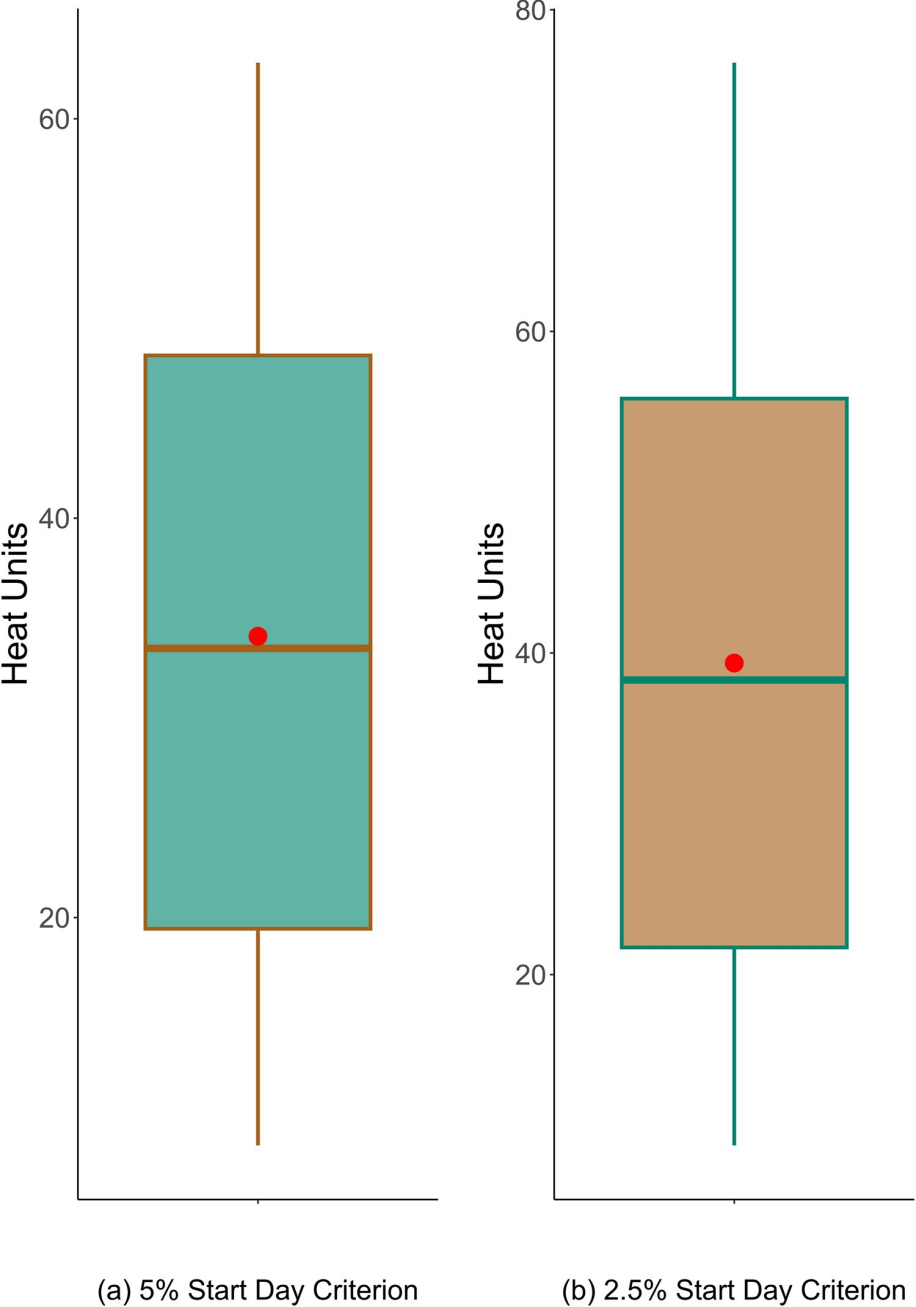
The box plot of candidate heat units for the peak day after all filtration has been performed including removal of the entries whose relative standard deviation is higher than 50% across years. The red dots show *M*, the mean values that will be used as the required heat units.

Figs 5 and 6 show the distributions of the retained candidate forcing units for the start day and peak days, respectively.

With the estimated parameters, the model had MAEs of 2.3 and 2.1 days on the training set for the start day prediction under the 5% and 2.5% criteria, respectively. The training also identified the mean forcing units *M* for testing the values in prediction, which were 14.70 and 16.28 under the 5% and 2.5% start day criteria, respectively. For the peak day prediction, the model had MAEs of 1.5 and 1.3 days for the two criteria, where the mean forcing units *M* were identified as 34.08 and 39.07 for the 5% and 2.5% start day criteria, respectively.

Then, these parameters were applied to the test years to assess the model performance on unseen data (Table 1).

**Table 1:** The optimal parameter values along with mean average errors (MAEs) for phenological modelling under the 5% and 2.5% criteria. Note that the optimal parameter values were derived from the training years while the MAEs indicate model performance on the test years.

Criteria	Start Day		Peak Day	
	Optimal Parameter Values	MAE (Days)	Optimal Parameter Values	MAE (Days)
**5%**	*F*_1_ = 31^st^ Jan *d* = -0.25 *c* = 15.5°C	3.7	*T*_*D*_ = 16.0°C *T*_*h*_ = 11.0°C	3.7
**2.5%**	*F*_1_ = 2^nd^ Feb *d* = -0.25 *c* = 14.0°C	2.7	*T*_*D*_ = 15.0°C *T*_*h*_ = 12.0°C	4.0

## Discussion

In this paper, two methods to reliably predict the spring pollen season (the start and peak days) of *Broussonetia papyrifera* in Islamabad were investigated. The pollen dataset from PMD showed that there were two flowering periods of this plant: one in the spring and the other towards the end of the summer. A previous study conducted on paper mulberry in neighboring India at a similar latitude also supported two flowering periods [24]. However, we focused on predicting the start and peak days of the spring pollen season because it causes significantly higher pollen concentrations than the summer flowering season (Fig 1).

### Strengths and limitations of the study

Two different modelling approaches based on regression and phenology to predicting pollen seasons were investigated and compared. The linear regression models were more accurate than phenological models for both the start and peak day predictions. These results are summarised in Table 2, which show that phenological models predicted the start day with an error of about 4 days, while the linear regression’s error is about 2 days when the start day is defined as the day on which 5% of the annual pollen is accumulated. The linear regression models and phenological models predicted the peak day with errors of about 3 or 4 days, respectively under the 5% start day criteria.

**Table 2:** Mean Absolute Error (MAE) for the start and peak days of the testing years using the linear regression and phenological modelling approaches under the 5% and 2.5% start day criteria of main pollen season (MPS).

Modelling Method	MPS Definition	Start Day (MAE)	Peak Day (MAE)
*LR*	5% − 95%	2.3	3.3
	2.5% − 97.5%	2.3	3.7
*PM*	5% − 95%	3.7	3.7
	2.5% − 97.5%	2.7	4.0

The accuracy of our models benefited from the longer period of modelling data (fifteen years), covering a wider range of weather conditions for the training and testing of the models, compared with previous studies. Moreover, we used leave-one-out cross validation, adjusted *R*^2^ and *p* value to reduce the chances of over-fitting our models.

The main uncertainty of this study is that the thermal requirements of the paper mulberry tree are unknown and hence in the phenological modelling we had to search a large sample space to identify the forcing units that were assumed to be specific to paper mulberry. This may explain the lower accuracy of our phenological models compared with our linear regression models.

### Correlate of findings with previous literature

Several studies have predicted the timing of pollen seasons [10]. One study predicting *Alnus* pollen concentrations for multiple cities in Spain used ARIMA time series regression models [25]. Another study [22] proposed models for eight pollen types. Their prediction model performed satisfactorily only for *Ulmus*, *Betula* and *Pinus* pollens, with differences between actual and predicted dates varying between 3–6 days. A study from Denmark [21] focused on predicting the start day of the pollen season for *Alnus*, *Betula* and *Ulmus* using pollen data from 1977–1990. Their prediction errors were between 2–4 days for *Alnus*, 3–5 days for *Betula*, and 8–10 days for *Ulmus*. In a recent paper, Picornell et al. proposed a model to predict the start day, peak day and end day of the main pollen season for the *Betula* tree in Bavaria, Germany [16]. Their model average error was 8.8 days for the start day, 3.6 days for the peak day and 3.8 days for the end day. Picornell et al. also reported an average error of 3.2 days for *Platanus* pollen season [23] and 2.6–3.5 days [26] for *Olea* pollen season by using a novel model called PhenoFlex. Therefore, our model predictions are similar or more accurate than the reported results.

Moreover, to the best of our knowledge there was only one paper that predicted pollen concentrations of paper mulberry, using data of paper mulberry concentration for the years 2003–2007, with the first four years used for training the model and the last year for testing the model [2]. This highlights the need for our study because climate change may have influenced the pollen season of paper mulberry in Islamabad. Using more recent and longer period of historical data for model training allows our model to be more robust under a wider range of weather conditions, which are more representative for the current climate.

The derived linear models suggest that temperature was the most relevant variable for predicting the start of the flowering. Previous studies also indicated that temperature was the most important parameter in determining the start of pollination for locations near the latitude 30°N (Islamabad is 33.7°N) [24]. Similarly, we estimated the daily temperature thresholds as 11–16°C, which were close to an average temperature of 19.4°C when flowering was observed in Chandigarh, India [24]. However, compared to Chandigarh, where flowering occurred in February—March, our data indicated that spring flowering of paper mulberry in Islamabad occurred in March—April. This delay in flowering was probably because Islamabad was slightly colder, i.e., the annual average temperate in Islamabad was 20.3°C while the annual average temperature in Chandigarh was 23.2°C.

### Implications for clinicians and policymakers

Exposure to high concentrations pollen of some plants is known to cause allergy symptoms in many individuals [27, 28]. One such plant species is the paper mulberry tree (*Broussonetia papyrifera*), whose high concentration is associated with increased risk of hospital visits due to asthma attacks [29] and severe respiratory problems in a large number of residents of Islamabad, Pakistan [7].

Patients and their healthcare providers generally use their personal experience and memory to predict pollen dates. However, these dates can vary significantly from one year to another. One can also construct a pollen calendar by averaging the pollen concentration on each calendar day so the patients can have a more reliable estimate of when to expect respiratory problems [30]. However, these pollen calendars are static in nature and do not consider variations in the weather from one year to another. We do not think these empirical and pollen calendar-based approaches are accurate enough for both patients and the healthcare system. The continuous availability of pollen and weather data along with the ubiquitous presence of internet and mobile phone makes it possible to provide more accurate predictions and raise timely alerts.

Since our algorithm was designed to start working on the 25^th^ of February each year, it gives enough time for people suffering allergy to take precautionary measures, such as reducing outdoor activities and starting required medication. To predict the start date is clinically important, as preventer medication takes several days to begin to be effective. Similarly, the peak date is critical for those at risk of severe, potentially life-threatening asthma attacks, who could be alerted via an SMS message or app of the risk in time to take preventive actions. Using technology like internet and text messages for asthma control have been valued in several studies and have found to be beneficial [31–36]. It is also important for health service planning. A few days may be required for an emergency department to ensure adequate staffing and equipment to cope with a sudden increase in acute asthma.

### Unanswered questions and future research

The most important question that arose during this study was about the temperature requirements, i.e., forcing units required for pollination to start. Despite extensive efforts, we could not find a study that observed the forcing units needed by paper mulberry to start flowering. Therefore, an in-situ study is needed to improve the accuracy of the phenological model. This will help understand the plant’s capability to flower in other territories or under other climatic conditions. This invasive plant is well known to have powerful mechanisms for asexual reproduction, so knowing the limits of its sexual reproduction could be useful to establish some control strategies.

The current study only investigated the environmental aspects of pollen maturation and shedding. To make it even more clinically significant, more research is needed to link individual responses to pollen exposure with personal factors such as concomitant (comorbid) disease, smoking, obesity, level of allergen sensitisation, and perhaps even living conditions, as well as average daily exposure to allergenic pollen particles.

The ultimate aim is to have patient-specific advice based on the patient’s clinical symptoms, allergy test results, and other aforementioned personal characteristics, as well as the predicted pollen season. An app could be built in collaboration with a healthcare provider to facilitate this. A randomized trial will be conducted to evaluate the effectiveness of using this app to prevent paper mulberry sensitised patients suffering from the pollen.

This work should be expanded to other geographical regions where pollen counters are not available and assess whether the weather parameters alone can successfully predict the pollen concentrations in those regions.

## Conclusion

In this work, two approaches aiming to predict the start and peak dates of the paper mulberry pollen season were designed and validated. The linear regression-based models performed better than the phenological models. Under the 5% criteria of the start day, linear regression model had mean absolute errors (MAEs) of 2.3 and 3.3 days for the start and peak days, respectively, while the phenological model had an MAE of 3.7 days for both the start and peak days. However, we anticipate that phenological modelling will perform better in years with unusual weather. Our findings could be used to provide information to patients and healthcare providers to start preparing for the paper mulberry pollen season. Timely action could avoid exposure, reduce the burden of symptoms, mitigate the risk of asthma attacks and potentially the prevention of deaths due to acute pollen induced allergy.
